# 固相萃取净化-超高效液相色谱-串联质谱法测定畜肉中18种卡因类麻醉剂

**DOI:** 10.3724/SP.J.1123.2022.08019

**Published:** 2023-05-08

**Authors:** Shaoming WU, Liqun OUYANG, Peng MENG, Menghang HE, Qin LIN, Yankai CHEN, Wenjing LIU, Xiaoming SU, Ming DAI

**Affiliations:** 福建省产品质量检验研究院食品检验研究所,国家加工食品质量检验检测中心,福建福州350002; Institute of Food Inspection, Fujian Inspection and Research Institute for Product Quality, National Quality Supervision and Testing Center for Processed Food, Fuzhou 350002, China

**Keywords:** 固相萃取, 超高效液相色谱-串联质谱, 卡因类麻醉剂, 畜肉, solid phase extraction (SPE), ultra-performance liquid chromatography-tandem mass spectrometry (UPLC-MS/MS), caine anesthetics, animal meat

## Abstract

随着麻醉剂被用于动物养殖、运输等领域,建立畜肉中麻醉剂残留的检测方法具有重要意义。该研究基于固相萃取-超高效液相色谱-串联质谱(UPLC-MS/MS)技术,建立了一种同时测定畜肉中18种卡因类麻醉剂残留的分析方法,实验通过优化18种卡因类物质的质谱参数,比较了在不同流动相中的分离度及响应强度,同时考察了不同提取条件、净化条件对18种卡因类物质提取率和净化效果的影响,结合外标法定量,实现了对畜肉中18种卡因类物质的定量分析。样品以0.1%(v/v)甲酸乙腈提取,Oasis PRIME HLB小柱净化,净化液经二甲亚砜辅助氮吹至近干,残渣用1.00 mL乙腈-水(1∶9, v/v)复溶,以0.1%(v/v)甲酸水溶液(含0.02 mmol/L乙酸铵)-甲醇作为流动相,经HSS T_3_色谱柱(100 mm×2.1 mm, 1.8 μm)分离,在电喷雾离子源(ESI)、正离子扫描和多反应监测(MRM)模式下进行UPLC-MS/MS测定。在最优条件下进行方法学验证,18种化合物在1.00~50.0 μg/L范围内线性关系良好,相关系数均大于0.999;检出限(LOD)为0.2~0.5 μg/kg,定量限为0.6~1.5 μg/kg;以猪肉、牛肉、羊肉为基质,进行3个不同水平的加标试验(*n*=6), 18种目标物的平均回收率为83.4%~100.4%,相对标准偏差(RSD)为3.1%~8.5%。该方法具有简单、快速、灵敏度高等优势,适用于畜肉中18种卡因类麻醉剂残留的快速测定,可为我国食品安全部门监控畜肉中卡因类麻醉剂残留提供技术支撑。

## Abstract (en)

Because of the widespread application of anesthetic drugs in the fields of animal breeding and transportation, demand for the rapid, sensitive detection of anesthetic drugs in animal meat is increasing. The complex animal meat matrix contains various interfering substances, such as proteins, fats, and phospholipids, along with anesthetic drug residues at very low concentrations. Therefore, adopting appropriate pretreatment methods is necessary to improve the sensitivity of detection. In this study, a rapid, accurate analytical method based on ultra-performance liquid chromatography-tandem mass spectrometry (UPLC-MS/MS) and solid phase extraction (SPE) was established to determine the contents of 18 caines in animal meat. The MS parameters, such as the collision energies of 18 caines, were optimized. Furthermore, the chromatographic separation conditions and response intensities of the caine in different mobile phases were compared. The effects of different pretreatment conditions on the extraction efficiencies of the 18 caines in meat samples and those of different purification conditions, such as extraction solvent, SPE column, and dimethylsulfoxide (DMSO) dosage, on their recoveries were investigated. Combined with the external standard method, the 18 caines in meat were successfully quantified. Sample pretreatment is a three-step process. First, in ultrasound-assisted extraction, 2.0 g samples were added to 2.0 mL water and extracted using 10 mL 0.1% (v/v) formic acid in acetonitrile under ultrasound conditions for 10 min. SPE was then performed using an Oasis PRIME HLB column. Finally, DMSO-assisted concentration was employed: the organic layer was collected and dried at 40 ℃ under a stream of N_2_ gas with the addition of 100 μL DMSO. Acetonitrile-water (1∶9, v/v) was added to the residue to yield a final volume of 1.0 mL for use in UPLC-MS/MS. The 18 caines were separated using an HSS T_3_ (100 mm×2.1 mm, 1.8 μm) column with 0.1% (v/v) formic acid in water (containing 0.02 mmol/L ammonium acetate) and methanol as mobile phases. Samples were detected using an electrospray ion source (ESI) in the positive ion and multiple reaction monitoring (MRM) modes during UPLC-MS/MS. Under the optimized conditions, the 18 target caine anesthetics displayed good linearities in the range of 1.00-50.0 μg/L, and the correlation coefficients (*R*^2^) were >0.999. The respective limits of detection (LODs) and quantification (LOQs) were 0.2-0.5 μg/kg, and 0.6-1.5 μg/kg. In pork, beef, and mutton samples, the recoveries obtained at three spiked levels were 83.4%-100.4% with relative standard deviations (RSDs) of 3.1%-8.5%. This simple, rapid, sensitive method may be applied in the detection of 18 caine anesthetics in animal meat and may provide technical support to the food safety department in China in monitoring the residues of caine anesthetics in animal meat.

卡因类化合物具有麻醉动物中枢神经的作用^[1,2]^,且麻醉速度快、复苏时间短^[3]^,是目前临床应用较为广泛的麻醉剂。近年来,一些养殖户在畜类出售之前将该类药物注入动物体内,使其进入麻醉状态,再将大量水注入,从而提高重量;另有一些商贩在运输途中,使用该类药物以减少动物应激性反应,降低死亡率,从而获得更高的经济收入。其中最常用的是三卡因,该化合物已被广泛用于活体运输领域,同时也是欧盟、加拿大、美国唯一允许使用的活体麻醉剂,但也有严格的限制,美国限定休药期为21天,并规定最大残留限量(maximum residue limit, MRL)为1 mg/kg^[4]^;加拿大限定休药期为5天^[5]^,挪威限定休药期为21天^[6]^。研究表明,卡因类麻醉剂具有中枢神经毒性以及心血管毒性^[7⇓-9]^,若畜类中滥用该类化合物,最终将会通过食物链进入人体,对人们的身体健康造成一定的威胁。我国GB 31650-2009标准《食品安全国家标准 食品中兽药最大残留限量》中没有该类化合物的相关限量,也没有相应的食品安全检测标准以及监管方式,因此,建立一种同时测定畜肉中多种卡因类麻醉剂的快速、准确、高灵敏度分析方法具有十分重要的现实意义。

目前对于卡因类药物的研究主要针对的是化妆品、药品、血液、水产品等基质,而对于畜肉中的研究鲜有报道。主要的检测方法有高效液相色谱法^[10⇓⇓⇓⇓⇓-16]^、高效液相色谱-质谱联用法^[17⇓⇓⇓⇓⇓-23]^、气相色谱-质谱法^[24⇓-26]^、离子色谱法^[27]^等。其中高效液相色谱法灵敏度低,且容易受到干扰,导致定性定量不准确;气相色谱-质谱法前处理过程复杂,效率低;离子色谱法同样存在易受干扰等问题;而液相色谱-串联质谱具有更高的灵敏度、更强的抗干扰能力以及更简单的前处理方式,已成为目前食品检测行业中广泛应用的分析方法。已有研究中,样品前处理主要采用固相萃取技术,根据化合物的性质,大多采用HLB固相萃取柱,净化过程通常需要活化、上样、淋洗、洗脱等步骤。近年来,Oasis PRIME HLB小柱采用保留杂质的通过式净化法,省去传统的活化、淋洗、洗脱步骤,可有效去除脂肪、磷脂等杂质,已在兽药残留检测领域中得到了广泛的关注和应用。

本研究采用超高效液相色谱-串联质谱法(UPLC-MS/MS),结合Oasis PRIME HLB固相萃取技术,建立了畜肉中18种卡因类麻醉剂的测定方法。该方法简单高效,灵敏度高,准确度和精密度好,适用于畜肉中卡因类化合物的测定。

## 1 实验部分

### 1.1 仪器与试剂

1290超高效液相色谱(安捷伦公司);AB SCIEX 5500三重四极杆质谱仪(AB SCIEX公司);Multifuge×4R Pro高速冷冻离心机(Thermo Fisher公司);Milli-Q超纯水纯化系统(Millipore公司);JT-C智能粉碎机(漯河金田公司);DS-8510 DTH超声波振荡器(上海分析超声仪器有限公司);MS 3 basic旋涡混均器(IKA公司); Oasis PRIME HLB固相萃取柱(3 mL/60 mg,Waters公司)。

普鲁卡因(procain, CAS号:59-46-1)、普鲁卡因胺(procainamide, CAS号:51-06-9)、氯普鲁卡因(chloroprocaine, CAS号:3858-89-7)、利多卡因(lidocaine, CAS号:137-58-6)、丙胺卡因(prilocaine, CAS号:721-50-6)、三卡因(MS-222, CAS号:886-86-2)、罗哌卡因(ropivacaine, CAS号:84057-95-4)、可卡因(cocaine, CAS号:50-36-2)、布比卡因(bupivacaine, CAS号:2180-92-9)、布他卡因(butacaine, CAS号:149-16-6)、丁卡因(tetracaine, CAS号:94-24-6)、苯唑卡因(benzocaine, CAS号:94-09-7)、普莫卡因(pramoxine, CAS号:637-58-1)、辛可卡因(cinchocaine, CAS号:85-79-0)、利索卡因(raythesin, CAS号:94-12-2)、间氨基苯甲酸(3-aminobenzoic acid, CAS号:99-05-8)、对氨基苯甲酸(4-aminobenzoic acid, CAS号:150-13-0)、对乙酰氨基苯甲酸(*p*-acetylamino benzoic acid, CAS号:556-08-1)单标准溶液(溶剂为甲醇)购自天津阿尔塔公司,质量浓度均为100 mg/L;甲酸、乙腈、甲醇(色谱纯,Merck公司),二甲亚砜(DMSO,分析纯,国药集团);所用水均为屈臣氏蒸馏水。

### 1.2 样品前处理

新鲜畜肉经组织粉碎机粉碎,放入-18 ℃冰箱保存,备用。解冻后准确称取样品2.0 g,加入2 mL水,再加入10 mL 0.1%(v/v)甲酸乙腈,涡旋后,超声波提取10 min,加入1 g氯化钠粉末涡旋后,以4500 r/min离心2 min,分取上层清液5.0 mL至Oasis PRIME HLB小柱净化,收集全部流出液,在流出液中加入100 μL DMSO后,于40 ℃水浴中氮吹至近干,残渣用1.00 mL乙腈-水(1∶9, v/v)溶液复溶,过0.22 μm滤膜后上机测试。

### 1.3 标准中间液及标准工作溶液的配制

准确吸取各单标准溶液(质量浓度均为100 mg/L)1.00 mL至100 mL容量瓶中,以甲醇定容至刻度,摇匀,配制成质量浓度均为1.00 mg/L的混合标准中间液,转入棕色瓶中,于-18 ℃保存,有效期为1个月。分别准确吸取混合标准中间液10、20、50、100、200、500 μL于6个10 mL容量瓶中,配制成质量浓度为1.00、2.00、5.00、10.0、20.0、50.0 μg/L的标准系列工作溶液。

### 1.4 色谱-质谱条件

#### 1.4.1 色谱条件

色谱柱:UPLC HSS T_3_柱(100 mm×2.1 mm, 1.8 μm, Waters公司);流动相:水相(A)为0.1%(v/v)甲酸水溶液(含0.02 mmol/L乙酸铵),有机相(B)为甲醇;流速为0.3 mL/min;柱温为35 ℃;进样量为2 μL;梯度洗脱程序:0~7 min, 5%B~30%B; 7~11 min, 30%B~80%B; 11~12 min, 80%B; 12.0~12.1 min, 80%B~5%B; 12.1~14 min, 5%B。

#### 1.4.2 质谱条件

电喷雾离子源(ESI):正离子扫描模式;多反应监测(MRM);离子化电压(IS): 5500 V;雾化气(Gas 1)压力: 3790 kPa(55 psi);辅助气(Gas 2)压力: 3790 kPa (55 psi);气帘气(CUR)压力: 2070 kPa (30 psi);离子源温度(TEM): 550 ℃;定性离子对、定量离子对及其他质谱参数见表1。

**表1:** 18种卡因类化合物的质谱参数

No.	Analyte	Precursor ion (*m/z*)	Product ion (*m/z*)	DP/ V	CE/ eV
1	3-aminobenzoic	138.1	77.0^*^	85	29
	acid		65.1	85	34
2	4-aminobenzoic	138.1	77.1^*^	60	30
	acid		65.1	60	19
3	procain	237.2	120.0^*^	40	20
			100.0	40	10
4	procainamide	236.1	163.1^*^	45	10
			120.2	45	10
5	*p*-acetylamino	180.1	94.1^*^	70	24
	benzoic acid		138.1	70	19
6	chloroprocaine	271.2	100.1^*^	46	22
			154.2	46	42
7	lidocaine	235.2	86.1^*^	40	23
			58.2	40	53
8	prilocaine	221.2	86.1^*^	40	20
			136.1	40	27
9	MS-222	166.1	138.1^*^	60	22
			94.0	60	30
10	ropivacaine	275.1	126.2^*^	60	27
			84.1	60	60
11	cocaine	304.2	182.1^*^	75	27
			150.2	75	34
12	bupivacaine	289.1	140.1^*^	60	28
			98.0	60	54
13	butacaine	306.2	178.1^*^	78	15
			120.1	78	15
14	tetracaine	265.2	176.2^*^	57	22
			72.1	57	40
15	benzocaine	166.1	138.1^*^	60	18
			94.0	60	24
16	pramoxine	294.2	100.2^*^	100	24
			128.1	100	29
17	cinchocaine	344.2	271.3^*^	75	30
			215.1	75	41
18	raythesin	180.1	120.0^*^	50	28
			94.1	50	24

* Quantitative ion; DP: declustering potential; CE: collision energy.

### 1.5 数据处理

采用Multiquant 4.0软件(AB公司)进行定量分析,Microsoft Excel 2016进行数据分析,OriginPro 9.1进行图谱处理。

## 2 结果与讨论

### 2.1 分析条件的优化

#### 2.1.1 质谱条件的优化

准确移取质量浓度为1.00 mg/L的混合标准中间液1.0 mL于5 mL容量瓶中,用50%甲醇水溶液定容至刻度,摇匀,配制成质量浓度均为200 μg/L的混合标准使用液,采用外置针泵连续进样模式、ESI源进行采集分析。大部分卡因类化合物属于酰胺类,分子结构中含有O、N等电负性较强的原子,具有较强的质子亲和力,容易得到质子形成准分子离子峰(母离子)^[21]^。因此,采用ESI^+^模式进行分析处理,先采用Q1扫描,分别获得[M+H]^+^母离子,再分别进行MS^2^(product ion)产物离子扫描,得到每个化合物的主要碎片离子,选取响应最大及次大的碎片作为定量离子和定性离子,再分别进行MRM扫描,优化去簇电压和碰撞能,优化后的质谱参数见表1。可以看出三卡因和苯唑卡因(9和15)、间氨基苯甲酸和对氨基苯甲酸(1和2)互为同分异构体,无法单靠质谱提取离子功能来区分,需要通过优化色谱条件将2对同分异构体完全分离。

#### 2.1.2 色谱条件的优化

对于卡因类化合物的测定,文献[17,21,28]采用了C_18_色谱柱。因此,本实验对比了常用的Waters Acquity UPLC BEH C_18_ (100 mm×2.1 mm, 1.7 μm)和Waters Acquity UPLC HSS T_3_ (100 mm×2.1 mm, 1.8 μm)色谱柱的分析效果。结果表明,HSS T_3_色谱柱对强极性化合物(间氨基苯甲酸保留时间为2.57 min)具有更好的保留能力(采用BEH C_18_时,间氨基苯甲酸保留时间仅为0.8 min),同时对于极性较弱的化合物(利索卡因、辛可卡因)也没有过强的保留,表现出更优的分离度,峰形也更尖锐。因此选择Waters Acquity UPLC HSS T_3_作为分析柱。由于采用的是ESI^+^模式,分别比较了0.1%(v/v)甲酸水溶液-甲醇、0.1%(v/v)甲酸水溶液-乙腈、0.1%(v/v)甲酸水溶液(含0.02 mmol/L乙酸铵)-甲醇、0.1%(v/v)甲酸水溶液(含0.02 mmol/L乙酸铵)-乙腈4种流动相体系对18种目标物峰形及响应的影响。结果显示,有机相为甲醇时,可以提高普鲁卡因、辛可卡因、丙胺卡因的响应,但布比卡因化合物色谱峰出现分叉;而含有乙酸铵时,布比卡因的峰形不再分叉,这是由于甲醇是质子供体,能够提高目标物的质子化效率,而乙酸铵是流动相改进剂,可以改善峰形^[18]^。因此,选择0.1%(v/v)甲酸水溶液(含0.02 mmol/L乙酸铵)-甲醇作为流动相,18种化合物的总离子流图见图1。包含的2对同分异构体(三卡因和苯唑卡因、间氨基苯甲酸和对氨基苯甲酸)也达到了基线分离。

**图1:**
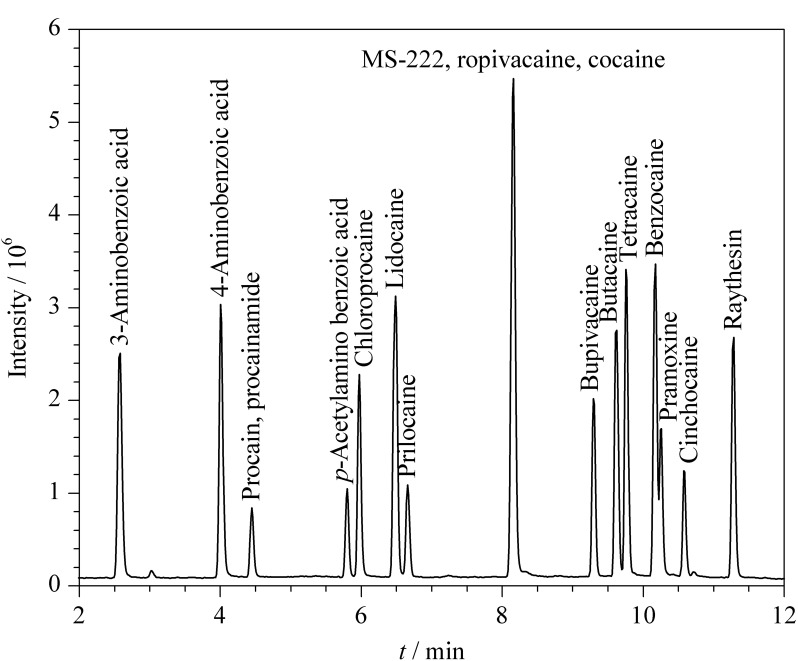
18种化合物的总离子流色谱图(20 μg/L)

### 2.2 样品前处理条件的优化

#### 2.2.1 提取溶剂的选择

卡因类化合物极性较强,文献报道的提取溶剂主要包括甲醇^[23]^、磷酸盐缓冲液^[29,30]^、0.1%(v/v)甲酸乙腈^[17,31]^和乙腈^[32]^。由于本实验选择的是保留杂质的净化方式,因此不考虑缓冲盐提取。实验选择空白猪肉样品进行10 μg/kg的加标回收试验,若直接采用有机溶液提取时,样品会结团,影响提取效率,因此先加入2 mL水使样品成为分散体系,再考察甲醇、乙腈、0.1%(v/v)甲酸甲醇、0.1%(v/v)甲酸乙腈作为提取溶剂的效果。结果见图2,采用甲醇或乙腈提取时,普鲁卡因胺、布比卡因和布他卡因的平均回收率为63.5%~67.5%,其余化合物的回收率均高于70%,与文献[11]结果一致;而采用0.1%(v/v)甲酸甲醇或0.1%(v/v)甲酸乙腈提取时,18种化合物的平均回收率为82.6%~98.2%;这是由于卡因类化合物大多属于伯胺类,加入甲酸有助于氨基的质子化,提高提取效率。此外,采用乙腈提取时,能更好地沉淀蛋白质,提取液更澄清,有助于后续的净化试验,综合考虑,采用0.1%(v/v)甲酸乙腈作为提取溶剂。

**图2:**
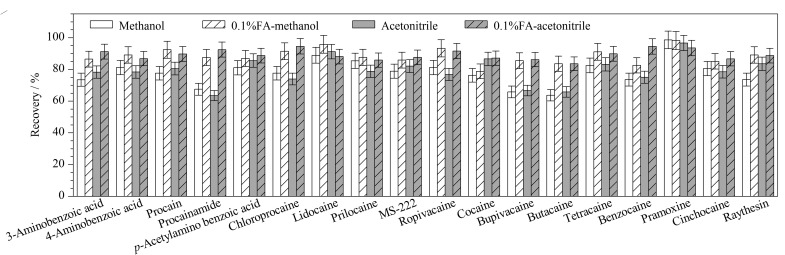
采用不同提取溶剂时18种化合物的回收率(*n*=3)

#### 2.2.2 净化条件的选择

由于0.1%(v/v)甲酸乙腈与水互溶,先加入1 g氯化钠粉末,再通过离心促使乙腈层和水相分离,可有效避免强极性的水溶性杂质进入提取液,导致仪器受到污染。此外,畜肉样品中含有大量的脂肪、蛋白质等杂质,同样会对仪器造成污染,还会造成基质效应,必须对提取液进行净化处理。对于卡因类化合物,文献主要采用 ${C_{18}}^{\left[23\right]}$ 、HLB^[29,31]^、MCX^[16]^固相萃取小柱净化,由于采用的是0.1%(v/v)甲酸乙腈提取,不宜采用MCX净化方式。因此,选用空白猪肉为基质,进行10 μg/kg的加标回收试验,对比了性质相近的C_18_(3 mL/60 mg,纳普公司)、PRIME HLB、HLB (3 mL/60 mg,Waters公司) 3种小柱通过式净化对结果的影响,结果见图3。

**图3:**
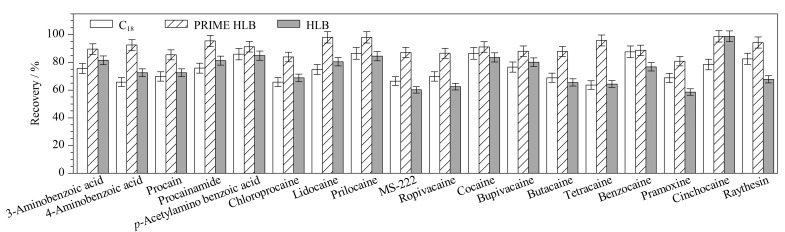
采用不同SPE小柱时18种化合物的回收率(*n*=3)

由图3可以看出,采用C_18_小柱净化时,5/18 (27.7%)的化合物平均回收率在80%以上;采用HLB小柱时,7/18 (38.9%)的化合物平均回收率在80%以上;而采用PRIME HLB小柱时,所有化合物的回收率均在80.9%~98.8%之间。此外,对比了采用3种净化方式净化后所得总离子流图的基线和干扰情况,使用PRIME HLB小柱所得的基线更低,杂峰更少,说明PRIME HLB小柱除了能更好地吸附杂质,对目标物的保留也更弱。因此,最终选择PRIME HLB小柱作为净化柱。

#### 2.2.3 DMSO用量的选择

由于净化后的溶液体积较大,且与流动相初始比例差距较大,会产生溶剂效应^[11]^,需将净化液浓缩后转换溶剂。试验发现,采用0.1%(v/v)甲酸乙腈配制的标准溶液在40 ℃下氮气吹干后,普鲁卡因、利多卡因、丙胺卡因、可卡因损失较大,回收率为56.7%~78.6%,其余化合物损失较小。而在做大批量样品时很难避免每个样品都不被吹干,考虑到DMSO与水、乙腈等都互溶,对于卡因类化合物的溶解性也较大,且沸点较高(189 ℃),尝试在浓缩前添加一定量的DMSO以防止吹干,并考察目标物的损失情况。试验了10、20、50、100、200 μL DMSO对结果的影响。结果表明,随着DMSO含量的增加,普鲁卡因、利多卡因、丙胺卡因、可卡因的损失逐渐降低,达到100 μL时基本无损失,回收率均在95.0%以上,因此选择DMSO用量为100 μL。

### 2.3 基质效应评价

使用质谱检测器会存在一定的基质效应(ME),导致目标物响应减弱或者增强,从而影响定量结果的准确性。以空白猪肉、牛肉、羊肉为基质,对方法的基质效应进行了考察,按照ME=(1-基质匹配标准溶液峰面积/溶剂标准溶液峰面积)×100%计算,ME在±20%以内表示基质效应不明显^[33]^。结果如图4所示,18种化合物在3种基质(猪肉、牛肉、羊肉)中均存在不同程度的基质抑制,ME为2.1%~8.0%,说明基质效应不明显,符合试验要求。

**图4:**
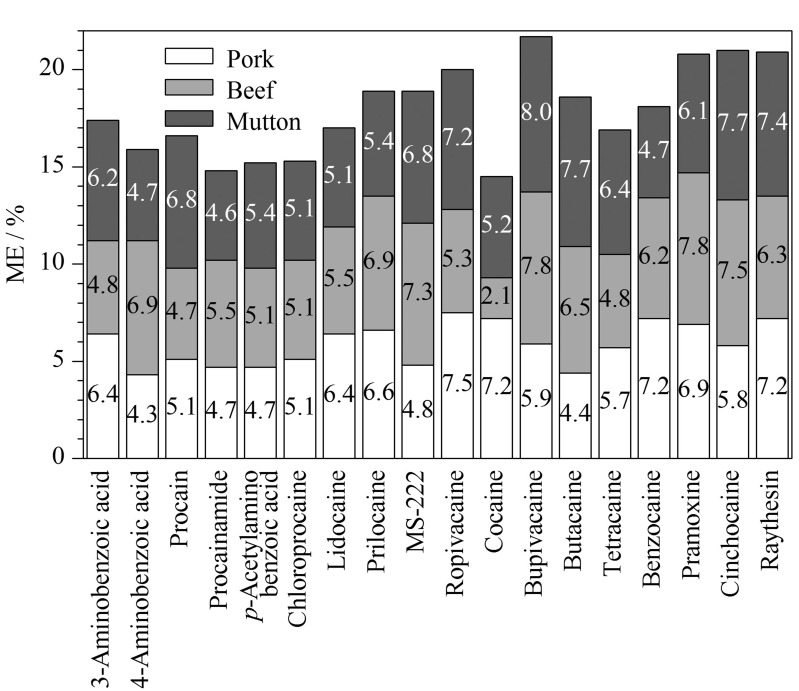
18种化合物在3种基质中的基质效应

### 2.4 方法学评价

#### 2.4.1 线性范围、检出限和定量限

将1.3节中配制的标准系列工作溶液上机测定,以各目标物的峰面积为纵坐标(*Y*),质量浓度为横坐标(*X*)绘制标准曲线,18种目标物在测定的范围内线性关系均较好,相关系数(*R*^2^)>0.999。此外,分别以空白猪肉、牛肉、羊肉为基质,进行1.00 μg/kg加标回收试验,以3倍信噪比和10倍信噪比确定检出限(LOD)和定量限(LOQ)。18种化合物在不同基质中的检出限为0.2~0.5 μg/kg,定量限为0.6~1.5 μg/kg(见表2),均低于文献[11,16,25,31]报道。

**表2:** 18种卡因类化合物的线性方程、相关系数、检出限和定量限

No.	Analyte	Linear equation	*R*^2^	LOD/(μg/kg)	LOQ/(μg/kg)
1	3-aminobenzoic acid	*Y*=6.7074×10^4^*X*+1.6754×10^4^	0.9997	0.2	0.6
2	4-aminobenzoic acid	*Y*=6.3861×10^4^*X*-8.6598×10^3^	0.9998	0.3	0.8
3	procain	*Y*=3.5157×10^4^*X*+3.8672×10^4^	0.9996	0.5	1.5
4	procainamide	*Y*=8.2482×10^3^*X*-4.8663×10^3^	0.9992	0.3	1.5
5	*p*-acetylamino benzoic acid	*Y*=2.0113×10^4^*X*-1.6876×10^4^	0.9998	0.4	1.3
6	chloroprocaine	*Y*=1.0525×10^5^*X*+7.8088×10^4^	0.9993	0.5	1.5
7	lidocaine	*Y*=2.0933×10^5^*X*+4.4610×10^5^	0.9991	0.3	0.8
8	prilocaine	*Y*=6.8383×10^4^*X*+2.7559×10^4^	0.9993	0.4	1.2
9	MS-222	*Y*=1.2682×10^5^*X*+1.6784×10^5^	0.9995	0.5	1.5
10	ropivacaine	*Y*=6.9264×10^4^*X*+2.8027×10^4^	0.9999	0.2	0.7
11	cocaine	*Y*=1.2682×10^5^*X*+1.6754×10^5^	0.9995	0.4	1.2
12	bupivacaine	*Y*=1.2092×10^5^*X*+1.3534×10^5^	0.9992	0.3	1.0
13	butacaine	*Y*=1.0196×10^5^*X*+3.3250×10^5^	0.9993	0.3	0.8
14	tetracaine	*Y*=1.8602×10^5^*X*+4.1724×10^5^	0.9991	0.2	0.6
15	benzocaine	*Y*=5.7538×10^4^*X*+7.9864×10^4^	0.9996	0.3	0.8
16	pramoxine	*Y*=2.9247×10^4^*X*+2.6919×10^4^	0.9995	0.5	1.5
17	cinchocaine	*Y*=4.9368×10^4^*X*+2.7170×10^4^	0.9994	0.5	1.5
18	raythesin	*Y*=1.0572×10^4^*X*+4.5010×10^3^	0.9995	0.4	1.0

*Y*: peak area; *X*: mass concentration, μg/L; linear range: 1.00-50.0 μg/L.

#### 2.4.2 准确度、精密度和稳定性

分别以空白猪肉、牛肉、羊肉为基质,进行2.00、5.00、20.0 μg/kg的加标回收试验,每个加标水平平行试验6次,计算各自的加标回收率和相对标准偏差(RSD),结果列于表3。18种化合物的平均回收率为83.4%~100.4%,RSD为3.1%~8.5%,说明该方法具有较好的准确度与精密度,满足GB/T 27404-2008 《实验室质量控制规范 食品理化检测》附录F的要求。此外,将同一加标水平(2.00 μg/kg)的样品溶液连续进样7天,计算回收率以及RSD,结果显示3种基质加标样品溶液中18种化合物的回收率为87.1%~97.8%,RSD为2.5%~5.1%,说明经该方法处理后的样品溶液在1周内稳定。

**表3:** 不同基质中18种卡因类化合物的回收率与精密度(*n*=6)

Analyte		Spiked/ (μg/kg)		Pork									Beef									Mutton					
				Recovery/%			RSD/%						Recovery/%			RSD/%						Recovery/%			RSD/%		
3-Aminobenzoic acid			2.00		91.5			5.8						88.7			4.4						83.4			5.6	
			5.00		92.3			3.9						90.9			6.3						93.0			4.3	
			20.0		88.6			4.6						91.2			4.3						93.8			6.2	
4-Aminobenzoic acid			2.00		96.9			4.3						95.5			5.0						90.0			4.2	
			5.00		94.6			4.3						90.5			4.6						98.5			4.9	
			20.0		90.5			4.6						89.2			4.6						96.1			4.6	
Procain			2.00		93.7			5.8						95.6			5.0						91.9			4.6	
			5.00		90.0			6.0						88.6			6.3						95.2			4.9	
			20.0		94.4			4.4						93.0			6.6						91.4			6.2	
Procainamide			2.00		92.6			6.8						91.2			4.8						95.9			6.5	
			5.00		85.6			6.5						89.8			7.4						94.1			4.7	
			20.0		89.5			5.4						88.2			7.1						87.0			7.3	
*p*-Acetylamino benzoic acid			2.00		89.6			4.0						88.3			5.9						90.9			7.0	
			5.00		93.5			5.2						92.1			4.4						91.0			5.8	
			20.0		96.7			6.5						95.3			5.6						95.0			4.3	
Chloroprocaine			2.00		88.9			6.3						89.7			7.1						98.3			5.5	
			5.00		93.0			5.3						91.6			6.8						90.3			7.0	
			20.0		87.9			6.5						86.6			5.7						94.5			6.7	
Lidocaine			2.00		91.2			6.8						92.5			7.1						89.3			5.6	
			5.00		96.3			4.8						94.9			7.4						92.7			7.0	
			20.0		92.5			3.1						91.1			5.2						97.8			7.3	
Prilocaine			2.00		88.6			4.0						87.6			3.4						94.0			5.2	
			5.00		95.3			5.4						93.9			4.4						90.0			3.4	
			20.0		97.8			5.5						89.9			5.9						96.9			4.3	
MS-222			2.00		92.6			4.6						91.2			6.0						99.3			5.8	
			5.00		97.6			3.1						93.6			5.0						94.1			5.9	
			20.0		93.6			7.5						92.2			3.4						99.2			4.9	
Ropivacaine			2.00		95.5			6.0						85.4			8.2						95.1			3.4	
			5.00		94.7			6.6						93.3			6.6						97.1			8.1	
			20.0		98.3			5.2						95.8			7.2						96.2			6.5	
Cocaine			2.00		91.0			4.6						89.6			5.6						99.8			7.1	
			5.00		88.9			5.8						97.7			5.0						92.4			5.5	
			20.0		94.7			7.2						93.3			6.3						90.3			4.9	
Bupivacaine			2.00		88.9			6.6						87.6			7.8						96.2			6.2	
			5.00		91.2			6.8						98.5			7.2						90.3			7.7	
			20.0		93.6			6.5						92.2			7.4						92.7			7.1	
Butacaine			2.00		98.7			5.5						87.9			7.1						95.1			7.3	
			5.00		95.5			6.2						94.1			6.0						100.3			7.0	
			20.0		91.1			4.9						89.8			6.7						97.0			5.9	
Tetracaine			2.00		97.7			6.8						96.3			5.4						92.6			6.6	
			5.00		93.3			4.3						96.6			7.4						99.3			5.3	
			20.0		92.4			5.2						91.0			4.6						94.8			7.3	
Benzocaine			2.00		86.6			5.7						89.9			5.6						93.9			4.6	
			5.00		88.9			7.6						91.5			6.2						88.0			5.5	
			20.0		92.3			5.9						90.9			8.3						90.3			6.1	
Pramoxine			2.00		95.6			6.5						98.9			6.4						93.8			8.2	
			5.00		94.7			4.8						93.3			7.1						97.2			6.3	
			20.0		98.3			6.0						96.8			5.2						96.2			7.0	
Cinchocaine			2.00		88.8			4.3						87.4			6.6						99.8			5.2	
			5.00		90.6			7.3						95.6			4.6						90.2			6.5	
			20.0		91.3			8.0						89.9			7.9						92.0			4.6	
Raythesin			2.00		98.8			6.5						94.6			8.7						92.7			7.8	
			5.00		93.9			7.2						92.5			7.1						100.4			8.5	
			20.0		94.4			3.1						93.0			7.8						95.4			7.0	

#### 2.4.3 与已报道方法的比较

与已报道的方法进行比较,结果见表4。本方法较液相色谱法^[11,16]^前处理用时更短,仅需23 min,消耗有机溶剂也更少,仅需10 mL,且灵敏度更高,检出限为0.2~0.5 μg/kg;相对于质谱法,文献[25,28,31,32]针对的都是水产品,最多涉及6个卡因类化合物,本方法针对的是畜肉样品,涉及18个卡因类化合物,灵敏度与文献[28,32]相当,高于文献[25,31],说明本方法具有较高灵敏度的同时,前处理用时更短、消耗有机溶剂更少,是一种快速、环保的前处理方法。

**表4:** 本方法与已报道的卡因类麻醉剂检测方法的比较

Samples	Analytes	Analytical methods	Preparation methods	Preparation time/min	*V*(Organic solvent)/mL	LODs/ (μg/kg)	Ref.
Fish and shrimp	three caines	HPLC-UV	DSPE	>30	10	11-43	[11]
Aquatic products	six caines	HPLC-UV	QuEChERS-SPE	>30	45	60	[16]
Aquatic products	tricaine	GC-MS/MS	SPE	>60	36	2	[25]
Aquatic products	tricaine and benzocaine	LC-MS/MS	SPE	>30	20	0.25	[28]
Aquatic products	six caines	LC-MS/MS	SPE	>30	10	1.5-6.0	[31]
Fish	five caines	LC-MS/MS	QuEChERS	23	10	0.3	[32]
Meat	18 caines	LC-MS/MS	pass-through SPE	23	10	0.2-0.5	this work

DSPE: dispersive solid phase extraction.

### 2.5 实际样品测定

采用新建立的方法,对本地市场中随机购买的10份猪肉、10份牛肉、10份羊肉进行18种卡因类麻醉剂残留进行测定,均未检出目标物。

虽然在实际样品中均未检测到18种卡因类麻醉剂残留,但实际样品的加标回收试验可证明本方法的准确性。

## 3 结论

本研究建立了一种PRIME HLB小柱净化-超高效液相色谱-串联质谱法测定畜肉中18种卡因类麻醉剂的分析方法。方法学验证表明所建立的方法具有良好的线性范围、较低的检出限、较高的准确度和精密度以及较小的基质效应,是一种快速、环保的分析方法,适用于畜肉中卡因类化合物的测定,可为食品安全部门对畜肉中卡因类麻醉剂的监管提供技术支持。
